# Theory and Application on Rough Set, Fuzzy Logic, and Granular Computing

**DOI:** 10.1155/2015/967350

**Published:** 2015-07-16

**Authors:** Xibei Yang, Weihua Xu, Yanhong She

**Affiliations:** ^1^School of Computer Science and Engineering, Jiangsu University of Science and Technology, Jiangsu 212003, China; ^2^School of Mathematics and Statistics, Chongqing University of Technology, Chongqing 400054, China; ^3^College of Science, Xi'an Shiyou University, Shaanxi 710065, China; ^4^Department of Computer Science, University of Regina, Regina, SK, Canada S4S 0A2

Recently, the rough set and fuzzy set theory have generated a great deal of interest among more and more researchers. Granular computing (GrC) is an emerging computing paradigm of information processing and an approach for knowledge representation and data mining. The purpose of granular computing is to seek for an approximation scheme which can effectively solve a complex problem at a certain level of granulation. This issue on the theory and application about rough set, fuzzy logic and granular computing, most of which are very meticulously performed reviews of the available current literature.

Four models of fuzzy or rough sets that are leading to a greater understanding of rough sets and fuzzy sets are discussed. These include multigranulation T-fuzzy rough sets, the so called approximation set of the interval set, the generalized interval-valued fuzzy rough set, and the *δ*-*cut* decision-theoretic rough set. Based on a kernelized information entropy model, an application on the fault detection and diagnosis for gas turbines is presented. The methods for reductions and their relevant algorithms are addressed in two manuscripts. Y. Zhang studies the distribution reduction in the inconsistent ordered information systems and further provides its algorithm. H. Ju et al. firstly give the model of *δ*-*cut* decision-theoretic rough set and then investigate the attribute reductions in this new decision-theoretic rough set model.

From the view of GrC, the optimistic multigranulation T-fuzzy rough set model was established based on multiple granulations under T-fuzzy approximation space by W. Xu. The manuscript of W. Li et al. improves the optimistic multigranulation T-fuzzy rough set deeply by investigating some further properties. And the relationships between multigranulation and classical T-fuzzy rough sets have been studied carefully. The interval set is a special fuzzy set, which describes uncertainty of an uncertain concept with its two crisp boundaries. Q. Zhang et al. review the similarity degrees between an interval-valued set and its two approximations and propose disadvantages of using upper approximation set or lower approximation as approximation sets of the uncertain set and present a new method for looking for a better approximation set of the interval set. T. Xue et al. also construct a novel model of the generalized fuzzy rough set under interval-valued fuzzy relation.

The aim of this special issue is to encourage researchers in related areas to discuss and communicate the latest advancements of rough set, fuzzy logic, and GrC, which covers both theoretical and practical results.

*Xibei Yang* *Xibei Yang* *Weihua Xu* *Weihua Xu* *Yanhong She* *Yanhong She*

